# Deer Blood Hydrolysate Protects against D-Galactose-Induced Premature Ovarian Failure in Mice by Inhibiting Oxidative Stress and Apoptosis

**DOI:** 10.3390/nu16203473

**Published:** 2024-10-14

**Authors:** Yu Wang, Hongyan Pei, Weijia Chen, Rui Du, Jianming Li, Zhongmei He

**Affiliations:** 1 College of Chinese Medicinal Materials, Jilin Agricultural University, Changchun 130118, China; wangyu102629@163.com (Y.W.); phy19990505@163.com (H.P.); chenweijia_jlau@163.com (W.C.); durui@jlau.edu.cn (R.D.); 2 Jilin Ginseng Academy, Changchun University of Chinese Medicine, Changchun 130117, China; 3 Key Laboratory of Animal Production, Product Quality and Security, Ministry of Education, Jilin Agricultural University, Changchun 130118, China

**Keywords:** deer blood hydrolysates, primary ovarian failure, oxidative stress, apoptosis

## Abstract

Background: Premature ovarian failure (POF) is a common disease among women, which can cause many complications and seriously threaten women’s physical and mental health. Currently, hormone replacement therapy is the primary treatment for premature ovarian failure. However, the side effects are serious and will increase the chance of breast cancer and endometrial cancer. Deer blood hydrolysate (DBH) is the product of enzymatic hydrolysis of deer blood, has antioxidant, anti-ageing, and anti-fatigue effects, and has the potential to improve premature ovarian failure. Methods: In our experiment, a mouse model of premature ovarian failure was established through intraperitoneal injection of 400 mg/kg/d of D-gal for 42 days. At the same time, different doses of DBH were gavaged to observe its ameliorative effect on premature ovarian failure. Results: The experimental findings indicated that DBH could restore the irregular oestrus cycle of POF mice, improve the abnormal amounts in serum hormones follicle-stimulating hormone (FSH), luteinising hormone (LH), progesterone (P) and estradiol (E2), increase the number of primordial follicles and decrease the number of atretic follicles. In addition, DBH also raised the level of superoxide dismutase (SOD) and reduced the level of malondialdehyde (MDA) and reduced the apoptosis of ovarian granulosa cells in mice. The WB assay results showed that gavage of DBH restored the decrease in the indication of nuclear factor erythroid 2-related factor 2 (Nrf2), Heme Oxygenase-1 (Ho-1), and B-cell lymphoma-2 (Bcl-2) proteins and reduced the elevated expression of Kelch-like ECH-associated protein 1 (Keap1), Bcl-2 associated X protein (Bax), and Cysteinyl aspartate specific proteinase-3 (Caspase-3) proteins that were induced by D-gal. Conclusions: To sum up, the present research indicated that DBH can ameliorate D-gal-induced oxidative stress and apoptosis by regulating the Nrf2/HO-1 signalling pathway and the Bcl-2/Bax/caspase-3 apoptosis pathway, which can be used for further development as a nutraceutical product to improve premature ovarian failure.

## 1. Introduction

Premature ovarian failure (POF) is regarded as ovarian failure taking place in women prior to reaching 40 years of age, with a prevalence of approximately 0.9–3% [1]. In addition to hair loss and night sweats, amenorrhoea and infertility, it also heightens the risk of osteoporosis and cardiovascular disease and triggers psychological problems, such as anxiety and depression, which seriously undermines women’s health [2,3]. The causes of POF are associated with multiple factors, including genetics, immunity, chemotherapy, and the environment. Currently, the main treatment for premature ovarian failure is hormone replacement therapy; however, it has serious side effects and heightens the occurrence rate of breast cancer and endometrial cancer [4,5].

The application of deer blood has a very long history in China, and it is documented in the Compendium of Materia Medica that deer blood has high medicinal value and health care function, which can prevent and treat a variety of diseases. In recent years, with the increasing awareness of health care, deer products have received more and more attention. However, most of the research focuses on antlers and venison, with less research and utilisation of deer blood, which is often discarded as a by-product of venison processing or made into cheap products, resulting in a certain waste of resources [6]. In fact, deer blood is rich in nutrients, such as proteins, lipids, free fatty acids, sterols, vitamins, etc., and contains a variety of macronutrients and beneficial trace elements, which have the effect of enhancing blood tonicity and enhancing immunity, as well as anti-ageing, and anti-inflammatory effects, which have a high research value [7]. Enzymatic hydrolysis is a common method of utilising animal proteins, and studies on the biological activities of enzymatically hydrolysis peptides are becoming more and more popular, such as antioxidant [8], antimicrobial [9], anti-inflammatory [10], anti-ageing [11,12], and angiotensin-converting enzyme (ACE) enzyme inhibition [13] activities. Deer blood hydrolysate (DBH), a product derived from the enzymatic digestion of deer blood, has been demonstrated to have an anti-fatigue effect, can effectively improve the swimming time of mice, has a good antioxidant and antibacterial ability, and also has the effect of regulating the intestinal microorganisms and intestinal injury in mice [14,15]; it also has the potential to improve premature ovarian ageing, but there has been no relevant study on this yet. In this experiment, we used intragastric administration; the enzymatic product, after digestion through the gastrointestinal tract, will produce some morphological and structural changes but still retain most of the bioactive functions. This method is the most common method of protein and peptide drug delivery, which has been adopted in many pieces of existing literature [16,17,18,19,20]. Our experimental results also validate the feasibility of this delivery method. Secondly, this method of administration has the highest public acceptance and can be easily developed into a health food product at a later date.

Therefore, in the current study, we investigated the protective function of deer blood hydrolysates against d-gal-induced ovarian POF in mice by detecting and analysing organ indices, oestrous cycle, histopathology, hormone levels, apoptosis of ovarian granulosa cells, and western blot experiments, and investigated the changes in the manifestation of oxidative apoptosis-associated proteins.

## 2. Materials and Methods

### 2.1. Materials

Deer blood powder was purchased from Shuangyang Deer Farm, Jilin, China. Alkaline protease, flavour protease and D-gal were purchased from Shanghai Yuanye Biotechnology Co., Shanghai, China. Mouse Luteinizing Hormone (LH) Quantitative ELISA Kit (YKE20085) and Mouse Follicle Stimulating Hormone (FSH) Quantitative ELISA Kit (YKE20084) were obtained from Huangshi Re-search Biotechnology Co., Huangshi, China. Mouse Estradiol (E2) ELISA Kit (JL11790) and Mouse Progesterone (PROG) ELISA Kit (JL20678) were obtained from Jianglai Science and Technology Co., Ltd. (Shenzhen, China). Superoxide Dismutase (SOD) Activity Assay Kit ( BC5165) and Malondialdehyde (MDA) Content Assay Kit (BC0025) were obtained from Beijing Solepol Technology Co., Beijing, China. The Hematoxylin–Eosin (HE) Stain Kit (G1120) was provided by Beijing Solepol Technology Co., Beijing, China. The Situ TUNEL Apoptosis Detection Kit was acquired from Beijing Biolabs Technology Co., Beijing, China (SNM535). The BCA Protein Quantification Kit (G2026) was purchased from Sevicebio Technology Co., Beijing, China. Anti-b-cell lymphoma-2 (Bcl-2, WL01556), an-ti-Bcl-2-associated X (Bax, WL01637), anti-caspase-3 (WL04004), anti-kelch-like ECH-associated protein 1 (Keap1, WL03285). Antibodies against nuclear factor e2-related factor (Nrf2, WL02135) and haemoglobin oxygenase-1 (HO-1, WL02400) antibodies were purchased from Shenyang Wanclass Biotechnology Co., Shenyang, China.

### 2.2. Preparation of DBH

Deer blood lyophilised powder was used as a raw material for enzymatic digestion using alkaline protease and flavoured protease in a ratio of 1:2. The lyophilised deer blood powder was prepared into a 9% solution with distilled water and then enzymatically digested for 4 hours at a temperature of 55 degrees Celsius and a pH of 8.5, and the added amount of protease was 6000 U/g. The enzyme solution was warmed at 90 degrees Celsius for 10 min. Then, it was centrifuged at 4000 rpm for 15 min, and the supernatant was lyophilised for spare parts.

### 2.3. Ascertaining the Amino Acid Composition of DBH

DBH was placed into sealable digestion tubes and hydrolysed with 6 mol/L HCl under vacuum, nitrogen filling and 110 °C for 22 h. Hydrochloric acid was removed through distillation, diluted to a certain concentration and then passed through 0.22 µm aqueous filtration membranes. An amino acid analyser (Hitachi, Tokyo, Japan) was used to analyse the amino acid composition.

### 2.4. Measuring the Molecular Weight of DBH

The molecular weight distribution of DBH was determined by high-performance liquid chromatography (HPLC) and analysed with GPC software (A.02.01) (Agilent, Santa Clara, CA, USA). The chromatographic column employed was TSKgel 2000 SWXL (300 mm × 7.8 mm) (Agilent, Santa Clara, CA, USA), and the mobile phase consisted of acetonitrile/water/trifluoroacetic acid (40:60:0.1, *v*/*v*/*v*). The samples were determined with a detection wavelength of 220 nm when the column temperature was 30 °C, and the flow rate was 0.5 mL/min. The standards were as follows: cytochrome C, peptidase, mycopeptide, ethionine-ethionine-ethionine and ethionine-ethionine-tyrosine-arginine.

### 2.5. Animals and Treatment

Mice were kept in standard laboratory environments at a room temperature of 23 ± 2 °C and a humidity of 60 ± 10% and had unrestricted access to food and water throughout the experiment. The experiment was approved by the Animal Care Professional Committee of Jilin Agricultural University, ethical review acceptance number: 2021 10 11 003.

Forty female C57BL-6 mice at 6–8 weeks and weighing 16–20 g were purchased from Changchun Yisi Experimental Animal Technology Co., Ltd. (Changchun, China) and were used in the experiments. According to previous experimental studies, we have determined that the low dose of DBH is 250mg/kg/d, the medium dose is 500 mg/kg/d, and the high dose is 1000 mg/kg/d. Administration of DBH has no toxic effect on mice. The mice were randomly assigned to five groups: a control group (CON), a POF group, and three DBH groups with varying doses of 250 mg/kg/day, 500 mg/kg/day and 1000 mg/kg/day. For each group other than the control group, 400 mg/kg of D-gal was injected intraperitoneally every day for 42 days, and the DBH-administered groups were gavaged with different dosages of DBH, while the control group was given the same volume of physiological saline. The body weight changes of the mice were recorded in the course of the experiment, and the oestrous cycle in mice was recorded from the 21st day. Using a cotton swab dipped in saline, mouse vaginal exfoliated cells were collected, applied to slides and stained with methylene blue (Yuan Ye Biotechnology Co., Ltd., Shanghai, China). When the experiment was over, mice were anaesthetised with sodium pentobarbital (Huaye Huanyu Chemical Company, Beijing, China), and blood was obtained by puncture while the uterus and ovaries of the mice were weighed and subjected to histological studies and biochemical analyses.

### 2.6. Enzyme-Linked Immunosorbent Assay (ELISA) Assay

Serum from mice was extracted by first allowing the blood to stand at room temperature for half an hour to clot. Then, it was centrifuged at 4 °C and 2000 revolutions per minute for 15 min to obtain the serum. According to the instructions of the commercially available ELISA kits, serum levels of FSH, LH, P, and E2, as well as levels of SOD and MDA in mouse ovarian tissues, were determined. The results were determined at a wavelength of 450 mm using an enzyme marker (BioTek, Shanghai, China).

### 2.7. H&E Staining of Ovary

The ovaries of mice were fixed for an entire night with 4% paraformaldehyde (Yuan Ye Biotechnology Co., Ltd., Shanghai, China). After that, they were dehydrated in ethanol, embedded in paraffin and sliced into 4 µm sections. Subsequently, hematoxylin–eosin (HE) staining was performed on the sections to analyse the morphological changes in the ovaries and uterus. 

### 2.8. TUNEL Analysis

First, ovarian sections were incubated with Proteinase K (20 mg/mL) for 25 min at 37 °C. Then, the sections were subjected to TUNEL assay using the In Situ Cell Death Detection Kit (Beijing Biolabs Technology Co., Beijing, China) according to the instructions. Apoptosis of ovarian granulosa cells was observed through a microscope. Live cells appeared blue, while apoptotic cells were brown. In apoptosis, chromosomal DNA double-strand breaks or single-strand breaks produce a large number of sticky 3′-OH ends. Under the action of deoxyribonucleotide terminal transferase (TdT), deoxyribonucleotides and derivatives formed by fluorescein, peroxidase, alkaline phosphatase, or biotin can be labelled to the 3′-end of DNA. Thus, apoptotic cells can be detected by TUNEL staining.

### 2.9. Western Blot Analysis

To observe mouse ovarian protein expression, we performed western blot experiments. Mouse ovaries were first lysed using RIPA lysis buffer (Wuhan Service Technology Co., Ltd., Wuhan, China) to extract the proteins, which were quantified. The electrophoresed proteins were transferred from the gel to a polyvinylidene difluoride (PVDF) membrane soaked in 5% skimmed milk powder for 2 h. The membrane was incubated overnight at 4 °C with the corresponding primary antibody. The membranes were then incubated with sheep anti-rabbit IgG secondary antibody for 2 h. After incubation of both primary and secondary antibodies, the membrane needs to be cleaned with pbst for good results: three times for ten minutes each. Chemiluminescence signals were detected and analysed using the Tanon 5200 Multi system from Tanon Biotechnology Co., Ltd. (Shanghai, China).

### 2.10. Statistical Analysis

All data were expressed as mean ± SEM. All experimental data were statistically analysed using Graphpad Prism 8.0 (GraphPad software, Inc., San Diego, CA, USA). The experimental data were analysed using SPSS 25, and all conformed to a normal distribution, the data were analysed by one-way or two-way ANOVA, Tukey’s test was used for comparison between multiple groups, and a *p* value of less than 0.05 indicated statistical significance.

## 3. Results

### 3.1. Amino Acid Composition and Molecular Weight Distribution of DBH

**Figure 1:**
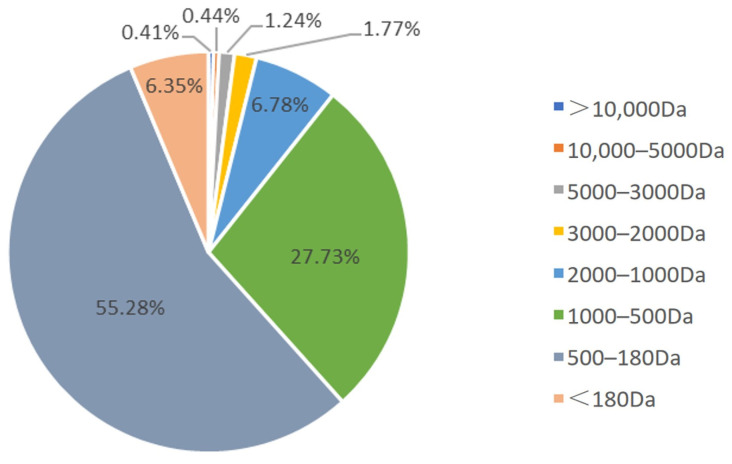
Molecular weight distribution of DBH.

As shown in Table 1, the results showed that DBH contained 17 amino acids at roughly 78.32 g/100 g. Leucine, aspartic acid, lysine, histidine, phenylalanine, valine, glutamic acid and alanine were the major amino acids in DBH. The Essential Amino Acid/Total Amino Acid (EAA/TAA) value of DBH was 44.6%, while the EAA/NEAA value was 80.45%, which was above 60%, suggesting that DBH can be utilised as a superior protein source. As shown in Figure 1, small molecular weight peptides of less than 1000 Da accounted for a large proportion of DBH, about 89.36%. The specific molecular weight distribution is as follows: 4.86 per cent for >2000 Da, 6.78 per cent for 1000–2000 Da, 27.73 per cent for 500–1000 Da, 55.28 per cent for 180–500 Da and 6.35 per cent for <180 Da. 

### 3.2. Effect of DBH on Body Weight and Ovarian and Uterine Indices in Mice

**Figure 2:**
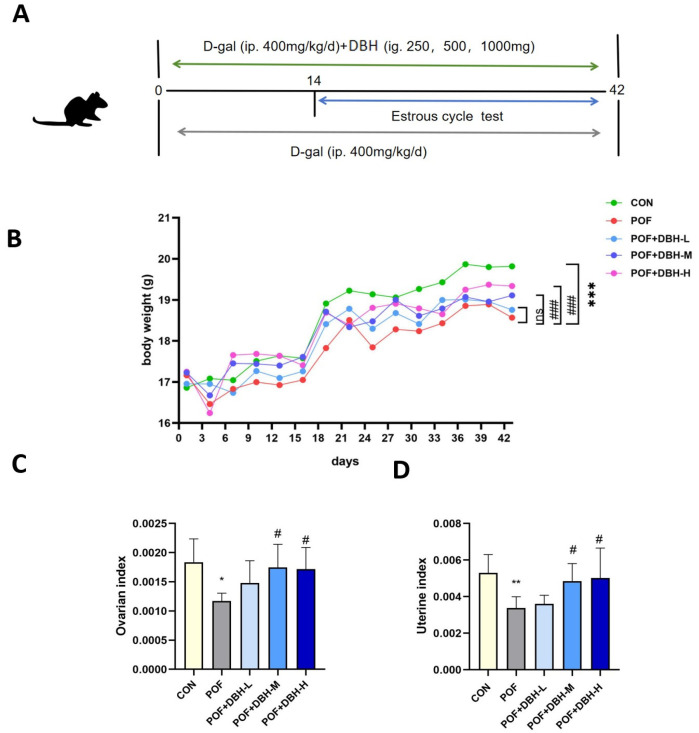
The experimental procedure and the impact of DBH on weight and ovarian and uterine indices in mice. (**A**) Diagrammatic illustration of the experimental procedure. (**B**) Changes in body weight of mice during the experiment. (**C**,**D**) Effect of DBH on ovarian and uterine indices. (mean ± SEM, *n* = 8, * *p* < 0.05 vs. control group, ** *p* < 0.01 vs. control group, *** *p* < 0.001 vs. control group, # *p* < 0.05 vs. POF group, ns *p* > 0.05 vs. POF group, ### *p* < 0.001 vs. POF group).

During the whole experiment, the mice did not die, ingested and excreted normally, and did not show abnormal behaviour. The effect of gavage DBH on the body weight and ovarian uterine index of mice was investigated by weighing them. As shown in Figure 2, the D-gal group showed a reduction in body weight in contrast to the control group. DBH treatment increased the weight of mice in a dose-related way as compared to the D-gal group. The ovarian and uterine indices of the POF group were significantly lower than those of the control group. However, in the DBH-administered group, these indices were elevated compared to the POF group. Moreover, the effect was significant in the medium- and high-dose groups. 

### 3.3. Effect of DBH on the Oestrous Cycle in Mice

**Figure 3:**
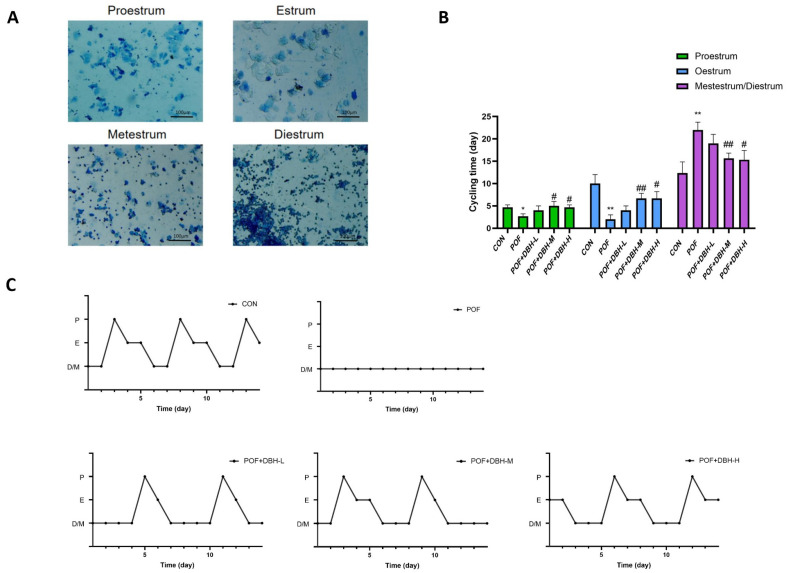
Effect of DBH on the oestrous cycle in POF mice. (**A**) Vaginal smears stained with methylene blue solution from different oestrus periods (P is preestrus, E is estrus, M is metestrus and D is diestrus). (**B**) Frequency of occurrence of cycle stages during the 27 days. (mean ± SEM, *n* = 8, * *p* < 0.05 and ** *p* < 0.01 vs. the control group; # *p* < 0.05 and ## *p* < 0.01 vs. the POF group). (**C**) Common patterns of regular and irregular estrus cycles.

To verify the effect of DBH on the oestrous cycle of mice with premature ovarian failure, we observed the variations in the oestrous cycle of mice after 21 days of drug administration by making vaginal smears and staining them (Figure 3A). The mice in the control group maintained a regular oestrus cycle, about 4–6 days a cycle, d-galactose induced mice oestrus cycle was disturbed, the oestrus cycle was disordered, and the inter-oestrus period was prolonged, and some of the mice showed oestrus cycle stagnation phenomenon. Some mice remained in the late stage of oestrus or the inter-oestrus period over a long period, and the administration of deer blood peptide to the group of each dose improved the oestrus cycle disorder of the mice to a certain extent and restored the regular oestrus cycle of the mice (Figure 3B,C). 

### 3.4. Effect of DBH on Serum Hormone Levels

**Figure 4:**
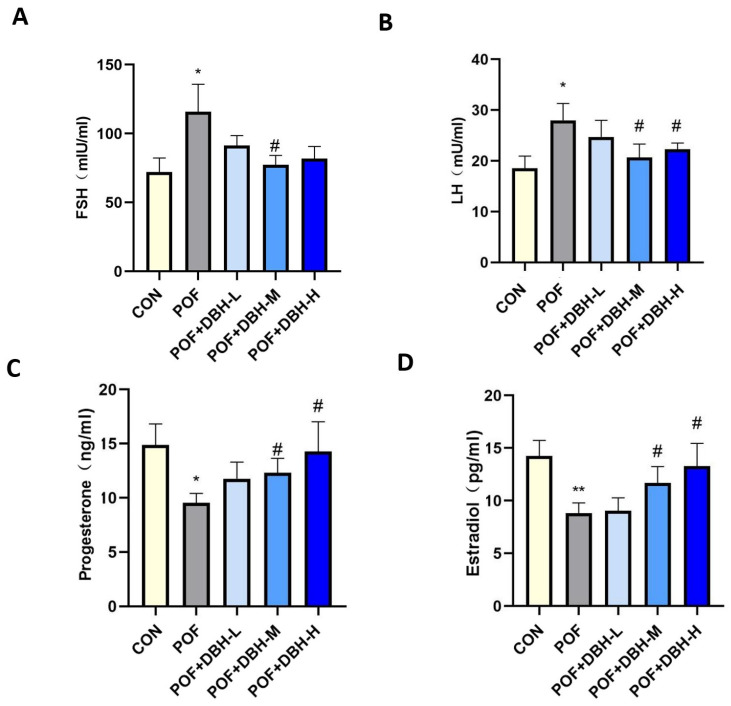
The impact of DBH on serum sexual hormones. (**A**–**D**) The FSH, LH, P and E2 levels of serum in different treatment groups. (mean ± SEM, *n* = 8, * *p* < 0.05 and ** *p* < 0.01 vs. the control group; # *p* < 0.05).

Serum hormone level is a crucial criterion for evaluating the ovarian status of mice, so we examined the levels of FSH, LH, P and E2 in the serum of mice. Figure 4 shows lower serum levels of FSH and LH in the POF group than in the control group and higher levels of P and E2. The application of DBH can, to some extent, eliminate the aforementioned changes in serum hormone levels, and the result is more remarkable in the medium- and high-dose groups.

### 3.5. Effects of DBH on Oxidative Stress

**Figure 5:**
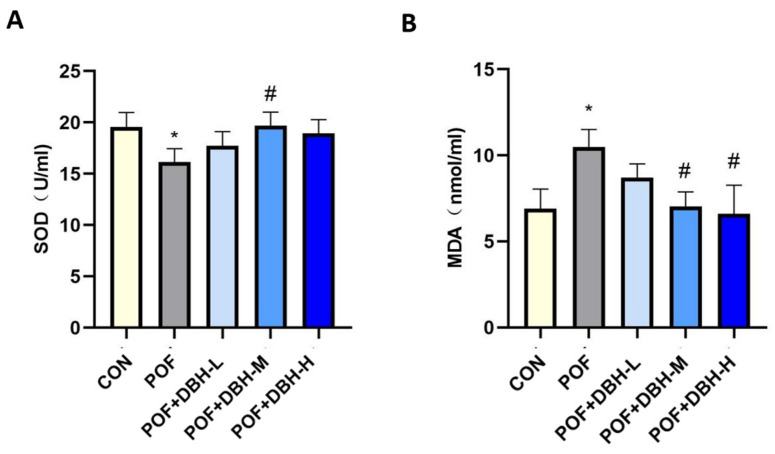
Effects of DBH on oxidative stress. (**A**) The SOD activity and (**B**) the MDA content were measured in the ovarian tissues. (mean ± SEM, *n* = 8, * *p* < 0.05 vs. the control group; # *p* < 0.05 vs. the POF group).

Oxidative stress is among the crucial causes of premature ovarian failure; SOD and MDA are significant indicators of the oxidative stress process. We examined the levels of SOD and MDA in mouse ovarian tissues. As shown in Figure 5, the results reveal that SOD activity was reduced in the POF group, as well as the level of MDA, was notably higher compared with those in the normal control group. SOD activity increased, and MDA levels decreased after treatment with DBH. This suggests that DBH can increase the activity of endogenous antioxidant enzymes for antioxidant purposes.

### 3.6. Effect of DBH on Ovarian Tissue Morphology and Follicle Number

**Figure 6:**
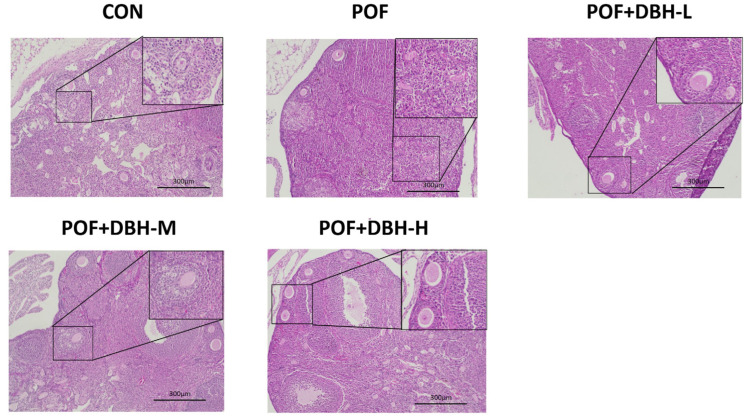
The ovaries were stained using H&E stain (100×) (*n* = 8).

Both ovarian structure and follicle number affect reproductive function in mice; we used HE staining of ovarian sections to assess the impact of DBH on ovarian structure and follicle development. As shown in Figure 6, follicles at all levels were visible in the blank group, with sufficient follicular reserve. The model group showed a reduction in the number of follicles at all levels and a rise in the number of atretic follicles, and the number of follicles at all levels increased after gavage of DBH, indicating that gavage of DBH can effectively increase the number of follicles in mice with premature ovarian failure, and has a protective effect on ovarian function.

### 3.7. Effects of DBH on Apoptosis in Ovarian Granulosa Cells

**Figure 7:**
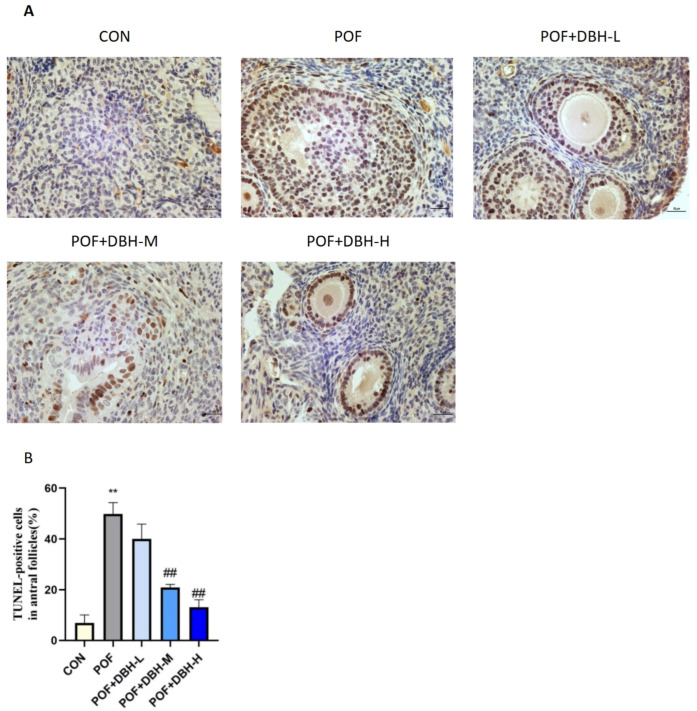
The impact of DBH on D-gal-induced ovarian cell apoptosis. (**A**): In situ TUNEL was used to analyse apoptosis. (200×) (**B**): Proportion of TUNEL-positive granulocytes to total cell count in different treatment groups. (mean ± SEM, *n* = 8, ** *p* < 0.01 vs. the control group; ## *p* < 0.01 vs. the POF group).

The apoptosis of ovarian granulosa cells was observed by using the tunel method. The results are shown in Figure 7, indicating that the proportion of apoptotic granulosa cells in the POF group was considerably higher compared to that in the control group. The proportion of apoptotic cells decreased significantly after gavaging with different doses of DBH, and the high-dose group had the optimal effect, indicating that the administration of deer blood peptide had the influence of improving the apoptosis of ovarian granulosa cells.

### 3.8. Effect of DBH on the Expression of Proteins in the Ovarian

**Figure 8:**
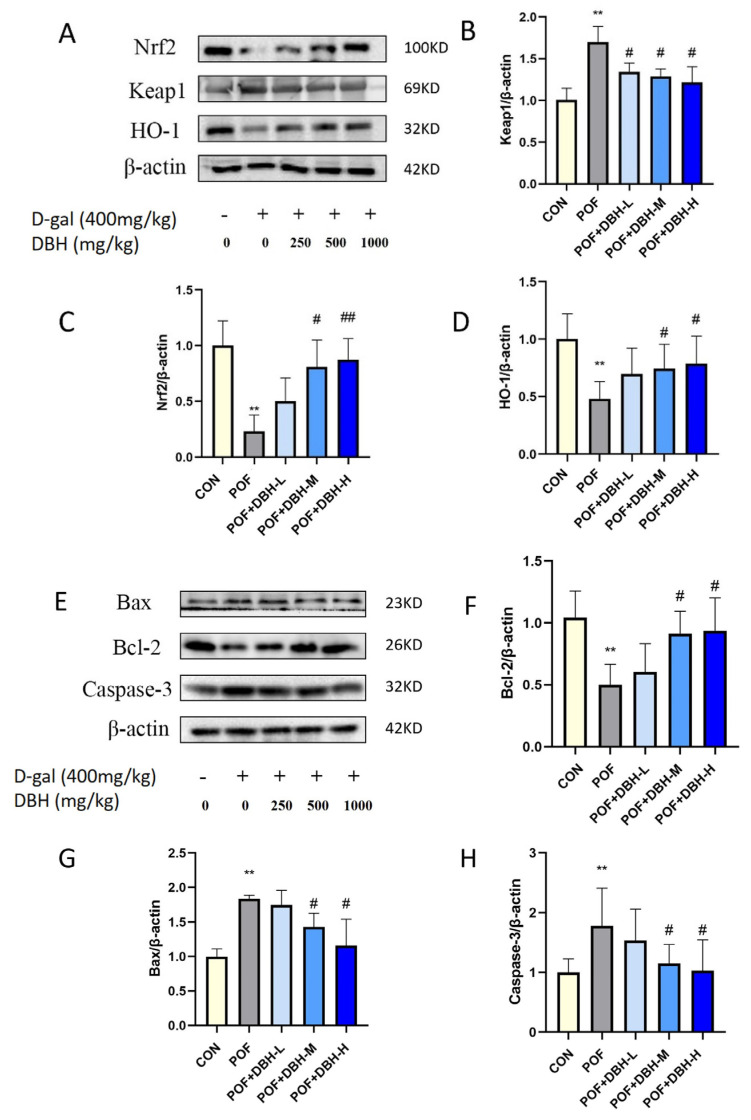
DBH’s effect on the expression of Nrf2/HO-1 and Bcl-2/Bax/caspase-3 within ovarian tissues. (**A**,**E**) Images that are representative of western blotting results. (**B**–**D**) Quantification of the expression of Nrf2, HO-1, and Keap1. (**F**–**H**) Quantification of the protein expression of Bcl-2, Bax, and caspase-3. (mean ± SEM, *n* = 3, ** *p* < 0.01 vs. the control group; # *p* < 0.05 and ## *p* < 0.01 vs. the POF group).

The expression levels of the proteins in ovarian tissues were analysed by immunoblotting. Compared to the control group, the expression of Keap1 protein was markedly up-regulated, while the expressions of Nrf2 and HO-1 proteins were significantly decreased, Bcl-2 protein expression was significantly reduced and Bax and caspase-3 protein expression was markedly enhanced in the POF group; these changes were reversed in the DBH administration group (Figure 8). This indicated that DBH could achieve the improvement of premature ovarian failure through anti-apoptosis and antioxidant influences, and its action was related to the signalling pathway of Bcl-2/Bax/caspase-3 and the signalling pathway of Nrf2/HO-1.

## 4. Discussion

Premature ovarian failure (POF) leads to amenorrhea, infertility, estrogen deficiency and increased gonadotropin concentrations in women under the age of 40 [21]. It is among the most frequently occurring primary hypogonadal disorders in women, with a prevalence of about 1 per cent among females [22]. Currently, the common treatment for POF is hormone replacement therapy. However, this therapy has side effects which raise the risk of breast cancer and heart attacks [23]. Nutritional supplementation through the consumption of foods with health benefits is one of the most effective ways to prevent and intervene in premature ovarian failure [24]. It has been demonstrated that taking proteins and peptides can improve premature ovarian failure, e.g., human lactoferrin [25], sea cucumber peptide [26], tilapia skin peptide [27], oyster polypeptide [28] and cockroach polypeptide of American cockroaches [29]. In the current study, deer blood was taken as raw material and enzymatically prepared to obtain deer blood hydrolysates to study its effect on D-gal-induced premature ovarian failure. 

D-gal is a commonly used method of modelling POF [30,31]. Disturbances in galactose metabolism lead to excessive accumulation of its intermediary products, resulting in a reduction in the number of ovarian follicles and ovarian granulosa cells and, consequently, in abnormal ovarian function [32]. D-gal also results in apoptosis of messy granulosa cells, increased atretic follicles, increased follicle-stimulating hormone (FSH) levels and decreased estradiol (E2) levels [33,34].

Our experimental results showed that POF mice had elevated serum levels of FSH and luteinising hormone (LH), decreased levels of E2 and progesterone (P), a decreased quantity of primordial follicles, and an increased number of atretic follicles. BDP administration elevated levels of E2 and P, decreased levels of FSH and LH and increased the number of follicles, and these results imply that BDP can function in ameliorating hormonal imbalances and restoring follicle number.

Reactive oxygen species (ROS) are produced during physiological and metabolic reactions in organisms and tissues. In normal circumstances, ROS act as cellular signal transduction molecules and exert an important regulatory function in the preservation of organismal homeostasis. Excessive oxidative stress is a major factor in causing POF [35]. Excess D-gal leads to an imbalance in the body’s oxidative and antioxidant mechanisms, which, in turn, leads to an overaccumulation of reactive oxygen species, thus inducing oxidative stress, which leads to follicle reduction, ovarian granulosa cell apoptosis and ovarian function impairment [36]. Superoxide dismutase (SOD) and malondialdehyde (MDA) levels are important indicators of the degree of oxidative stress [37]. SOD is a significant antioxidant enzyme that eliminates superoxide anion free radicals in the body and stops oxidative damage to cells [38]. MDA is a substance formed through the reaction between lipids and superoxide radicals and indicates the degree of oxidation [39]. In this experiment, it was observed that SOD content decreased and MDA content increased after D-gal injection, whereas administration of BDP elevated SOD content and decreased MSD content. This indicates that administration of DBH can improve the oxidative stress induced by D-gal. The Nrf2/HO-1 pathway exerts a significant role in the processes of antioxidation and anti-apoptosis [40,41]. When cells are attacked by ROS, Nrf2 detaches from Keap1 and rapidly translocates to the nucleus and forms a heterodimer with sMaf protein, which then binds to the antioxidant response element ARE, thereby transcriptionally activating the expression of antioxidant genes regulated by Nrf2 and exerting antioxidant effects, such as HO-1, to maintain the dynamic redox balance in vivo [42,43,44]. The results showed that the expression amounts of Nrf2 and HO-1 were significantly increased, and the Keap1 expression level was significantly decreased after DBH administration. This suggests that BDP has antioxidant capacity and can ameliorate D-gal-induced oxidative stress in POF mice by activating the Nrf2/HO-1 signalling pathway.

The granulosa cells are located on the exterior of the zona pellucida of the oocyte and are the basic unit of cells in the ovary. They take part in supporting and nourishing the oocyte and contribute to its growth. Apoptosis of the granulosa cells triggers the apoptosis of the oocyte, which, in turn, causes follicular atresia, leading to premature ovarian failure [45,46]. Therefore, reducing apoptosis of ovarian granulosa cells is also one of the most effective ways to improve premature ovarian failure. The expression of bcl-2 and bax genes in ovarian tissues is closely related to the apoptosis of primordial cells and oocytes, which can be used as an important basis for the determination of premature ovarian failure. Bcl-2 expression was significantly reduced in ovarian tissues of an animal model of POF, whereas the expression of bax was significantly increased [47,48,49]. We observed the apoptosis of mouse ovarian granulosa cells by tunel staining. The results showed that BDP significantly improved D-gal-induced ovarian granulosa cell apoptosis. In addition, we performed west blot experiments, and the results indicated that the expression of the anti-apoptotic factor bcl-2 was down-regulated, while the expressions of the pro-apoptotic factors bax and caspase-3 were up-regulated. These are induced by D-gal and could be reversed after DBH treatment, which suggests that BDP can attenuate D-gal-induced POF by inhibiting apoptosis and that this process is related to the Bcl-2/Bax/caspase-3 apoptotic pathway.

## 5. Conclusions

In summary, the results of the present study indicated that deer blood hydrolysate could ameliorate D-gal-induced oxidative stress and apoptosis by modulating the Nrf2/HO-1 signalling pathway and Bcl-2/Bax/caspase-3 apoptotic pathway. DBH also attenuated oestrous cycle disorders, hormonal imbalance and histopathological changes in POF mice. These results suggest that DBH can be further developed as a functional food for preventing POF and delaying the process of POF, a finding that not only facilitates the comprehensive use of deer blood but also provides a new option for patients with premature ovarian failure.

## Figures and Tables

**Table 1:** Amino acid composition of DBH (mg/g).

Name of Compound	Content [mg/g]	Retention Time [min]	Area of Peak [mV.s]
Aspartic acid	86.177	8.379	1157.193
Threonine	47.061	10.252	706.85
Serine	36.341	11.184	677.315
Glutamic acid	58.608	13.501	869.663
Glycine	31.305	19.783	946.61
Alanine	52.243	21.015	1265.785
Cystine	6.934	22.623	39.466
Valine	61.357	23.228	842.938
Methionine	10.296	25.167	149.9
Isoleucine	6.344	26.561	84.405
Leucine	88.818	27.463	1273.287
Tyrosine	27.03	29.877	253.724
Phenylalanine	62.717	30.755	703.877
Histidine	67.928	35.744	718.785
Lysine	72.651	36.971	845.813
Arginine	35.383	45.156	414.208
Proline	32.111	14.944	229.51
Total amount	783.304		11,179.33

## Data Availability

The research data employed to back up the findings of this study are included within the article.
